# Underlying mechanisms of long-term potentiation during the inhibition of the cannabinoid CB1 and GABAB receptors in the dentate gyrus of hippocampus

**DOI:** 10.1186/s12868-022-00767-z

**Published:** 2023-01-12

**Authors:** Masoumeh Nazari, Seyed Asaad Karimi, Somayeh Komaki, Masoumeh Kourosh Arami, Alireza Komaki

**Affiliations:** 1 Department of Physiology, School of Medicine, Hamadan University of Medical Sciences, Shahid Fahmideh Street, 65178/518 Hamadan, Iran; 2 Department of Neuroscience, School of Science and Advanced Technologies in Medicine, Hamadan University of Medical Sciences, Hamadan, Iran; 3 Department of Neuroscience, School of Advanced Technologies in Medicine, Iran University of Medical Sciences, Tehran, Iran

**Keywords:** Hippocampus, Paired-pulse ratio, Cannabinoid CB1 receptors, GABA_B_ receptors, Long-term Potentiation

## Abstract

**Background:**

The release of various neurotransmitters and thereby the excitability of neuronal circuits are regulated by the endocannabinoid system in an activity-dependent manner. Hippocampal long-term potentiation (LTP) is augmented in cannabinoid type 1 (CB1) receptor-deficient mice. CB1 receptors exist on GABAergic axon terminals in the hippocampus. In our previous work, we showed that CB1 antagonists increased the population spike (PS) amplitude, field excitatory post-synaptic potential (fEPSP), and the LTP induction in the dentate gyrus (DG) of the rat hippocampus while the GABA_B_ antagonist decreased these parameters. Determining the underlying mechanisms of the pre- and/or postsynaptic locus of LTP expression is of great importance. In this study, we investigated whether LTP alteration acutely caused by CB1 and GABA_B_ receptor antagonists (AM251 and CGP55845, respectively) happens at the postsynaptic or presynaptic regions, or at both. Therefore, the paired-pulse ratio (PPR) was assessed prior to and following the LTP induction in the studied groups.

**Methods:**

Male Wistar rats were randomly assigned to the groups of control, AM251, CGP55845, CGP55845 + AM251. A high-frequency stimulation (HFS) of the perforant path (PP) was used to induce LTP in the DG region.

**Results:**

Statistical analysis revealed that AM251 produced significant increase in excitatory postsynaptic potential (EPSP) slope and amplitude of PS. Conversely, administration of CGP55845 produced decrease in slope of EPSP. The current results indicated that the PPR was not influenced by LTP induction in the presence of AM251 or CGP55845 either alone or their combination.

**Conclusions:**

It can be concluded that the site causing LTP expression is, at least in part, the postsynaptic site because PPR was not influenced by LTP induction in the presence of AM251 or CGP55845 either alone or their combination.

## Background

LTP is a crucial neurochemical foundation of memory and learning and plays a role in the putative mechanism of memory formation and/or retrieval of memory in the mammalian hippocampus; a brain structure embedded deep in the temporal lobe [1]. Paired-pulse facilitation (PPF) as a kind of short-term synaptic plasticity (STP) is applied for probing the mechanisms of LTP [2]. STP is essential for cognitive and information processing in neuronal circuits, and represents a change in synaptic strength on time scales from hundreds of milliseconds to seconds and includes mechanisms for both facilitation of transmitter release (increase in synaptic strength), and depression (decrease in synaptic strength) [3, 4]. STP mechanisms predominantly are presynaptically and Ca^2+^-dependent, although postsynaptic mechanisms may also contribute [3].

Neuromodulators and neurotransmitters can exert effects on LTP and can change the threshold for induction of PPF and LTP [5, 6]. Earlier investigations clearly establish that the endocannabinoid and GABAergic systems play a role in the physiological mechanisms underlying memory and learning [7–9]. CB1 and CB2 have been cloned and identified for the endocannabinoid systems [10]. The cannabinoid CB1 and CB2 receptors can be considered as class a of G protein-coupled receptors (GPCRs). CB1 receptors are mainly distributed in the hippocampus, cerebral cortex, cerebellum, and basal ganglia [11, 12]. CB1 receptors are metabotropic and couple to the Gi/o family of heterotrimeric G-protein coupled receptors and decreasing Ca^2+^ influx, increase K^+^ efflux [12–14].

Endocannabinoids are vital negative regulators of transmitter release and impact synaptic plasticity [15]. Previous studies have revealed that cannabinoids through CB1 receptors inhibit LTP [16, 17]; or modify LTP [18]. In CB1-knocked out mice (CB1-KO), LTP was augmented in vitro and in vivo in the hippocampus [19, 20]. On the other hand, the endocannabinoid-induced reduction of inhibitory transmission facilitates hippocampal LTP by disinhibiting pyramidal neurons [21, 22]. These contradictory reports might be clarified by the fact that the CB1 receptor in the hippocampus regulates the release of both glutamate and GABA, two neurotransmitters with opposite impacts [23].

CB1 receptors are present in the different synaptic connections in the central nervous system (CNS), especially in the hippocampus [23] affect, releasing neurotransmitters, like glutamate or GABA in CNS [24, 25]. GABA is the principal inhibitory neurotransmitter in the CNS, which is formed by the decarboxylation of glutamate [26]. GABA_A_, GABA_B_, and GABA_C_ receptors have been cloned and identified for GABAergic systems [26]. The GABA_B_ receptors are metabotropic and belong to the family of heterotrimeric G-protein coupled receptors. When activated, these receptors inhibit adenylyl cyclase and increase Cl^–^ influx and K^+^ efflux and inhibit Ca^2+^ influx [26]. GABA_B_ receptors can mediate both postsynaptic and presynaptic inhibition [26].

There are different results on the effects of endocannabinoid and GABAergic systems on LTP and STP. For instance, AM251 as a selective antagonist of the CB1 receptor can inhibit LTP induction in the dendritic area of the CA1 neurons and induce retrograde amnesia in rat models [27]. Conversely, in our previous work we show that AM251 increased the LTP induction in DG of rat hippocampus [28]. There are functional relations and reciprocal inhibition between the CB1 and GABA_B_ receptors in hippocampal membranes [29]. Also, it has been reported that there is overlapping anatomical localization between the CB1 and GABA_B_ receptors in the CNS, especially in the hippocampus [29–31].

To understand how the CB1 receptor-mediated regulation of GABAergic transmission involved in the fine-tuning of LTP, in the current study we assessed hippocampal LTP in the presence of CB1 and GABA_B_ receptors antagonist alone and in a combination with both.

Determining the underlying mechanisms of pre- and/or postsynaptic locus of LTP expression is of great importance. Here we investigated whether LTP induction caused by CB1 and GABA_B_ receptors antagonist is mediated at the postsynaptic or presynaptic neuron, or at both. Accordingly, we assessed the PPR of two responses evoked by two successive stimuli at certain intervals, since alterations at the presynaptic regions can alter the PPR [3].

## Methods

### Ethics statement

All experimental procedures using rats were conducted in accordance with the animal care and use guidelines approved by the institutional ethics committee at Hamadan University of Medical Sciences (Ethics Code: IR.UMSHA.REC.1392.11.15) and also the National Institutes of Health Guide for Care and Use of Laboratory Animals. The research was also conducted based on the ARRIVE guidelines. We tried to minimize animals suffering and operations causing distress and pain were performed in a separate room.

### Animals and experimental design

Adult male Wistar rats (220 ± 10 g) were purchased from animal breeding colony of Hamadan University of Medical Sciences, Hamadan, Iran. The rats were kept in a room with a temperature of 22 ± 2 °C with under a 12-h light/dark cycle in cages (2–3 animals per cage). Standard animal chow and water were provided for the animals. After adaptation for one week, the rats were randomly allocated to the groups (n = 8–10) of control (90% saline + 10% DMSO), AM251, CGP55845, CGP55845 + AM251.

### Chemicals and drugs administration

We used the 4-methyl-1-H-pyrasole- 3-carboxamide (AM251) as a synthetic CB1 receptor antagonist prepared from Sigma, USA and the GABA_B_ receptor antagonist (2S)-3-[[(1S)-1-(3,4-dichlorophenyl) ethyl]amino-2-hydroxypropyl](phenylmethyl) phosphinic acid hydrochloride (CGP55845; Sigma, USA). CGP55845 and AM251 were first dissolved in DMSO (dimethylsulfoxide) (Sigma, USA), followed by diluting in saline (0.9% NaCl). The concentration of DMSO was < 10%. DMSO and saline were applied as the vehicle. Microinjection of the drugs into the DG was done through a 27-gauge stainless steel injector that was attached to Hamilton microsyringe (1 µl) by PE-20 tubing about 20 min before HFS. Therefore, 0.5 µl of the drugs was microinjected unilaterally using a microsyringe pump and Hamilton syringes at 0.5 µl/min for 1 min. After combination of the drugs, their amount was twice and their final amount was the same (0.5 µl). We left the injection syringe in position for 60 s prior to withdrawal for minimizing dragging of the administrated solutions along the injection tract. The used solutions were prepared promptly before their usage. The concentrations of the drugs was according to earlier investigations: AM251: 0/1 µg/rat [28, 32, 33], and CGP55845: 1 µg/rat [28, 34].

### Stereotaxic surgery and LTP recording

**Fig. 1:**
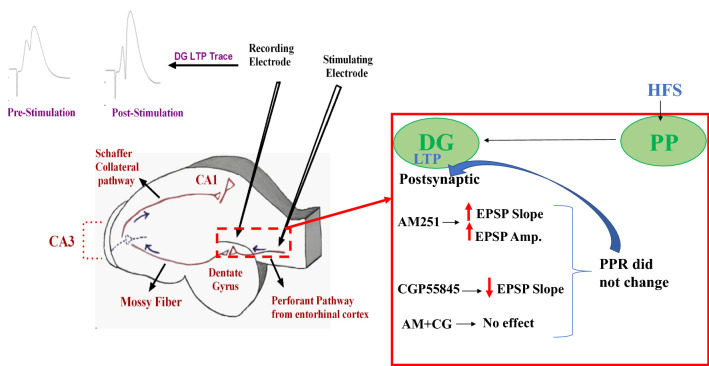
Representative graph indicating sites of stimulating and recording electrodes in a hippocampus sagittal section. Representative sample traces of evoked field potential in the DG noted before and 60 min following high frequency stimulation are indicated at the top of the figure (left side). Hippocampus figure was painted by Dr. Seyed Asaad Karimi

**Fig. 2:**
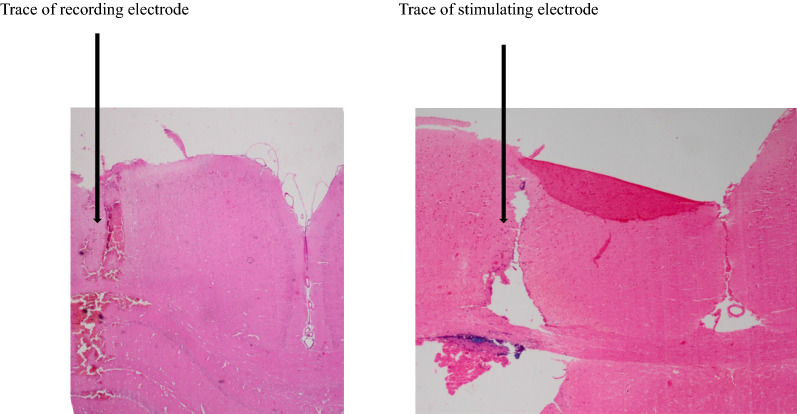
Exemplary photomicrograph illustrating places of stimulating and recording electrodes in a hippocampus section. Stimulating and recording electrode traces can be seen at the right and left sides, respectively. Scale bar: 0.5 mm

Surgical procedure and hippocampal LTP experiments were performed essentially as described in our previous works [35]. After anaesthetization with urethane (1.5 g/kg) the rats were located in a stereotaxic apparatus for recording and surgery. During surgical procedure, the rats were positioned on a thermostatically controlled heating blanket and their core body temperature was evaluated through a thermometer inserted into their rectum and preserved at 37.0 ± 0.2 °C. After exposing the skull, we placed a concentric bipolar stainless-steel stimulating wire electrode (insulated with Teflon (no tips); 125 μm diameter) in the lateral perforant path (LPP) based on the atlas of Paxinos and Watson with the following coordinates: 4.3 mm lateral to the midline, 8.1 mm posterior to bregma, 3.2 mm ventral below the skull surface [36]. Also, we lowered a bipolar recording electrode with coordinates of 2.3 mm lateral to the midline and 3.8 mm posterior to bregma) into the DG until observing the maximum fEPSP (2.7–3.2 mm ventral). Through electrophysiological monitoring of the DG response following single-pulse PP stimulation, the optimal ventral placement was achieved (Fig. 1). The electrodes were lowered gently (0.2 mm/min) from the cortex to the DG, for reducing brain tissue injury [37]. Figure 2 is indicating locations of stimulating and recording electrodes.

Through variation in the intensity of the SPS as well as averaging five responses per intensity, an input/output (I/O) response curve was built. The intensity of a stimulus evoking an average field potential of 50% of the maximal response was employed for all next stimulations. Following determination of the I/O curves, we used single stimuli presented every 10 s for 30 min, and monitored the responses. The fEPSP slope was measured from 20 to 80% of the peak amplitude. fEPSP slopes were normalized considering a control period of 30 min prior to the tetanic stimulation. As soon as a stable baseline of responses was achieved during 20 min, LTP induction was done using a 400 HZ HFS protocol (0.2 ms stimulus duration, 10 bursts of 20 stimuli, 10 s interburst interval) at a stimulus intensity evoking a field EPSP slope and PS amplitude of about 50% of the maximal response. PS and EPSP were recorded 60 min following HFS for determining the DG neurons’ synaptic responses. At all-time points, 10 continuous evoked responses were averaged at stimulus intervals of 10 s [38, 39].

We used our software (eTrace, www.sciencebeam.com) for defining the stimuli parameters and they were transferred to an A365 constant-current isolator unit produced by World Precision Instruments using a data acquisition board before they were delivered to the PP by the stimulus electrode. For amplification (1000 × ) (Differential amplifier DAM 80, World Precision Instruments), and filtration (band pass 1 Hz to 3 kHz), obtained field potential response was passed through a preamplifier and then was digitized at 10 kHz and observed by an oscilloscope and a computer. The data were stored in an electronic file for further offline assessments.

### Measurement of evoked potentials

Measurement of evoked potentials were performed essentially as described in our previous works [8]. fEPSP and PS are two main components of evoked field potential in the DG. Assessment of the PS amplitude was done from the initial positive deflection peak of the evoked potential to the next negative potential peak. The field EPSP slope function was calculated using the slope of the line that connected the beginning of the initial positive deflection of the evoked potential to the second positive deflection peak of the evoked potential. The intensity of the stimulation was fixed at evoke potentials comprising 40% of the maximum population spike amplitude, described using an input/output curve [35, 37].

### Paired-pulse stimulation (PPS)

The short-term plasticity was investigated using PPS that was sent to the DG every 10 s at interpulse intervals of 3 ms. In all experiments, the interstimulus interval (ISI) was different systematically from 20 to 40 ms. Hence, a full series contained a paired pulse sequence characterized by the ISIs of 20, 30 and 40 ms. For calculating the PPR, the second response amplitude of the pair was divided by the respective first response amplitude. For each rat, a minimum of 10 consecutive ISI series were measured and averaged [35]. PPR was assessed prior to and following LTP induction. After the experiments, the rats were sacrificed by high doses of ketamine.

### Statistical analysis

Values are described as mean ± SEM and analyzed by GraphPad Prism® 8.0.2. The groups were compared by Two-Way ANOVA followed by Sidak’s. A P-value smaller than 0.05 was regarded significant. The LTP value was calculated using the following formula:

$$\mathrm{LTP}=\frac{\mathrm{the \,EPSP \,or\, PS \,value\, after\, HFS \,induction }\times 100\mathrm{\%}}{the\, average\, EPSP \,or\, PS \,at\, baseline}$$

## Results

### Effects of AM251 or CGP55845 either alone or their combination on the EPSP slopes of DG granular cells

LTP recordings were achieved in the DG granular cells after PP stimulation. HFS applied to the PP-DG area could induce LTP in treated rats. Figure 1 shows the representative graph indicating locations of stimulating and recording electrodes.

**Fig. 3:**
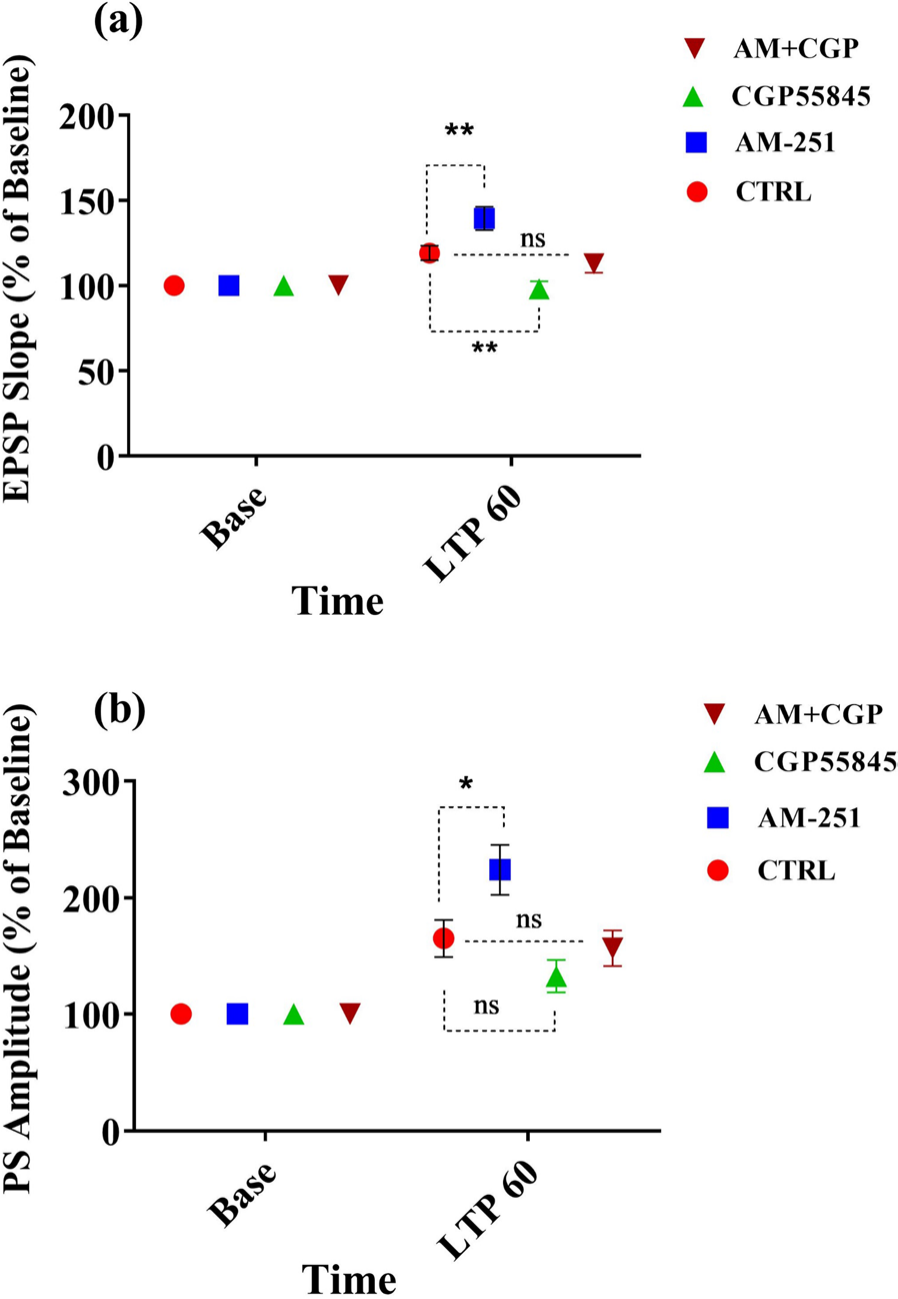
Time-dependent alterations in hippocampal responses to perforant path stimulation after a HFS. The effect of AM-251 (CB1 receptor antagonist), CGP (GABA_B_ receptor antagonist), and AM + CGP on LTP induced by high frequency stimulation to performant path. The graphs show the changes of slope (**a**) and amplitude (**b**) of fEPSP recorded from DG neurons compared to baseline (control, CTRL). Values are reported as mean ± SEM % of baseline. *P < 0.05, **P < 0.01, *ns* not significant

A Two-Way ANOVA was employed for indicating the variability among the groups. A significant effect of time [F (1, 72) = 42.87, P < 0.0001] and treatment [F (3, 72) = 10.36, P = 0.0001] in EPSP slope of DG the granular cells (Fig. 3a) was observed. Statistical analysis using Two-Way ANOVA followed by Sidak’s test revealed that AM-251 produced significant increase in slope of EPSP (p < 0.01, Fig. 3a). Therefore, blockade of CB1 receptors augments the slope of EPSPs in DG granular cells by HFS of the perforant path. It means that cannabinoids may decrease the LTP induction in DG cells.

In addition, we evaluated the changes of fEPSP slope during HFS to PP by administration of GABA inhibitor, CGP55845 compared with the corresponding controls. Administration of CGP55845 produced decrease in slope of EPSP compared to CTRL group (p < 0.01, Fig. 3a). These results show that inhibition of GABAergic neurons reduce the slope of EPSPs in DG granular cells by HFS of the perforant path. Thus, GABAergic activity in DG region of hippocampus may potentiate the LTP induction in DG neurons.

The AM + CGP and CTRL groups showed no significant differences (p > 0.05). Thus, it seems that there is no effect of CB1 and GABA inhibition on LTP, because the potentiation effect of AM may be counteracted by the inhibitory effect of CGP on fEPSP slope.

### Effects of AM251 or CGP55845 either alone or their combination on the PS amplitude of DG granular cells

Two-Way ANOVA showed a significant effect of time- points [F (1, 72) = 67.66, p < 0.0001] and treatment [F (3, 72) = 5.316, p = 0.0023] in PS amplitude of the DG granular cells (Fig. 3b).

Statistical analysis using Two-Way ANOVA followed by Sidak’s test revealed that AM-251 produced significant increase in amplitude of PS (p < 0.01, Fig. 3b). Therefore, blockade of.

CB1 receptors increases the amplitude of EPSPs in DG granular cells by HFS of the perforant path. It means that cannabinoids may decrease the LTP induction in DG cells.

Administration of CGP55845 did not change amplitude of PS in compared with CTRL group (p > 0.05, Fig. 3b). Therefore, GABA transmission failed to exhibit a significant effect on amplitude of PS in DG granular neurons.

The AM + CGP and CTRL groups showed no significant differences (p > 0.05). It may be proposed that the excitatory effect of AM251 on amplitude of PS is mediated mostly by the cannabinoid receptors on GABAergic neurons. Therefore, inhibition of GABAergic receptors may counteract this excitatory effect.

### Impact of LTP induction on the PPR of evoked potentials

In the paired pulse procedure, we evaluate the amplitude of an initial EPSP with that of a second EPSP triggered shortly after the first EPSP. If the second EPSP is larger than that of the first EPSP, it is called paired pulse facilitation (PPF), whereas if the second EPSP is smaller, it is called paired pulse depression (PPD). The hypothesis for PPF is the accumulation of calcium ions and so more transmitter release. Thus, PPF may have a presynaptic origin. The depression of PPR is called PPD. If the PPR did not changed it shows the postsynaptically origin of synaptic plasticity [23].

**Fig. 4:**
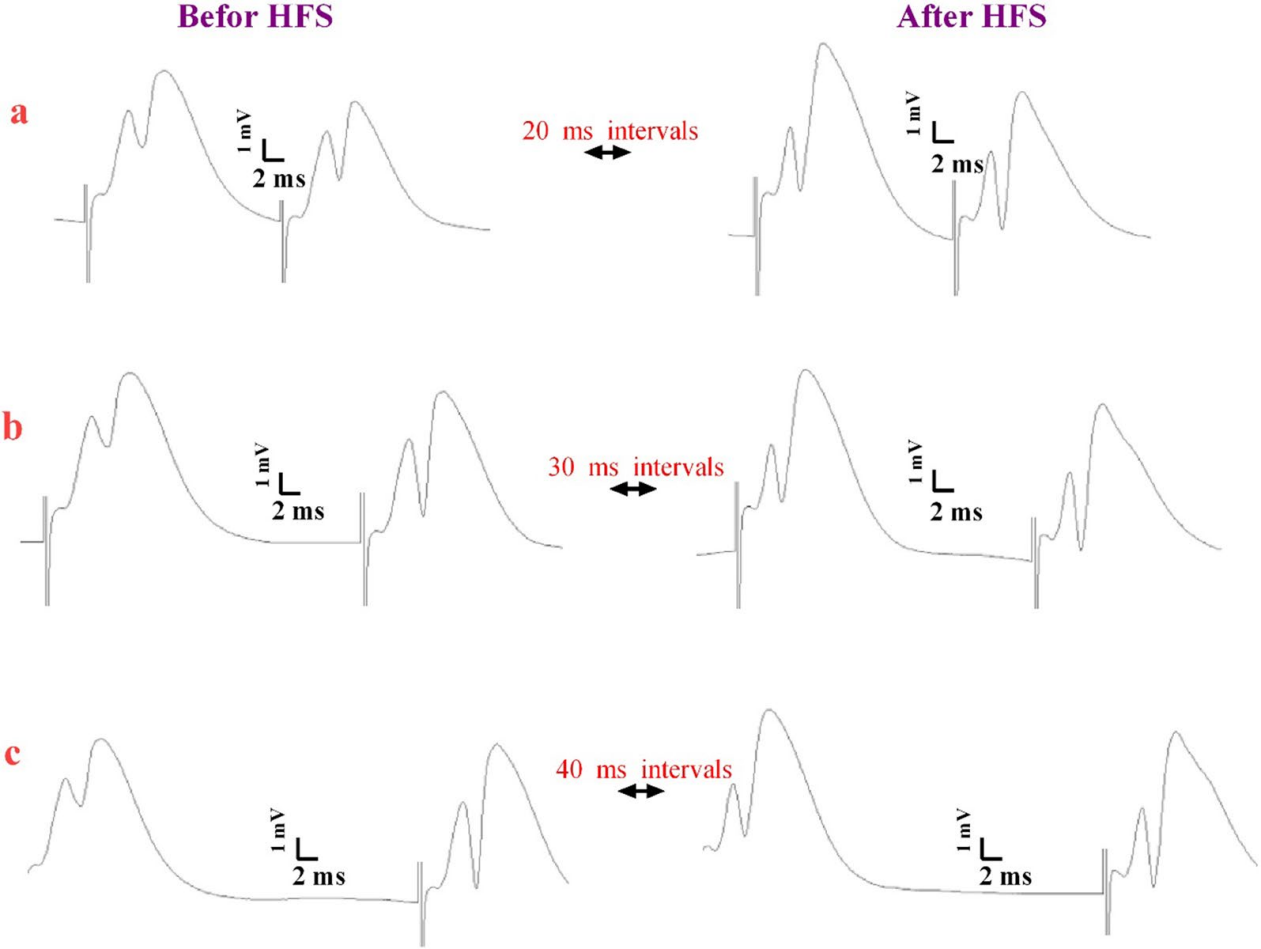
Representative sample traces of field potentials evoked by paired-pulse stimulation at intervals of 20 (**a**), 30 (**b**) and 40 (**c**) ms before and after high-frequency stimulation in the hippocampal PP-DG pathway in male rats

**Fig. 5:**
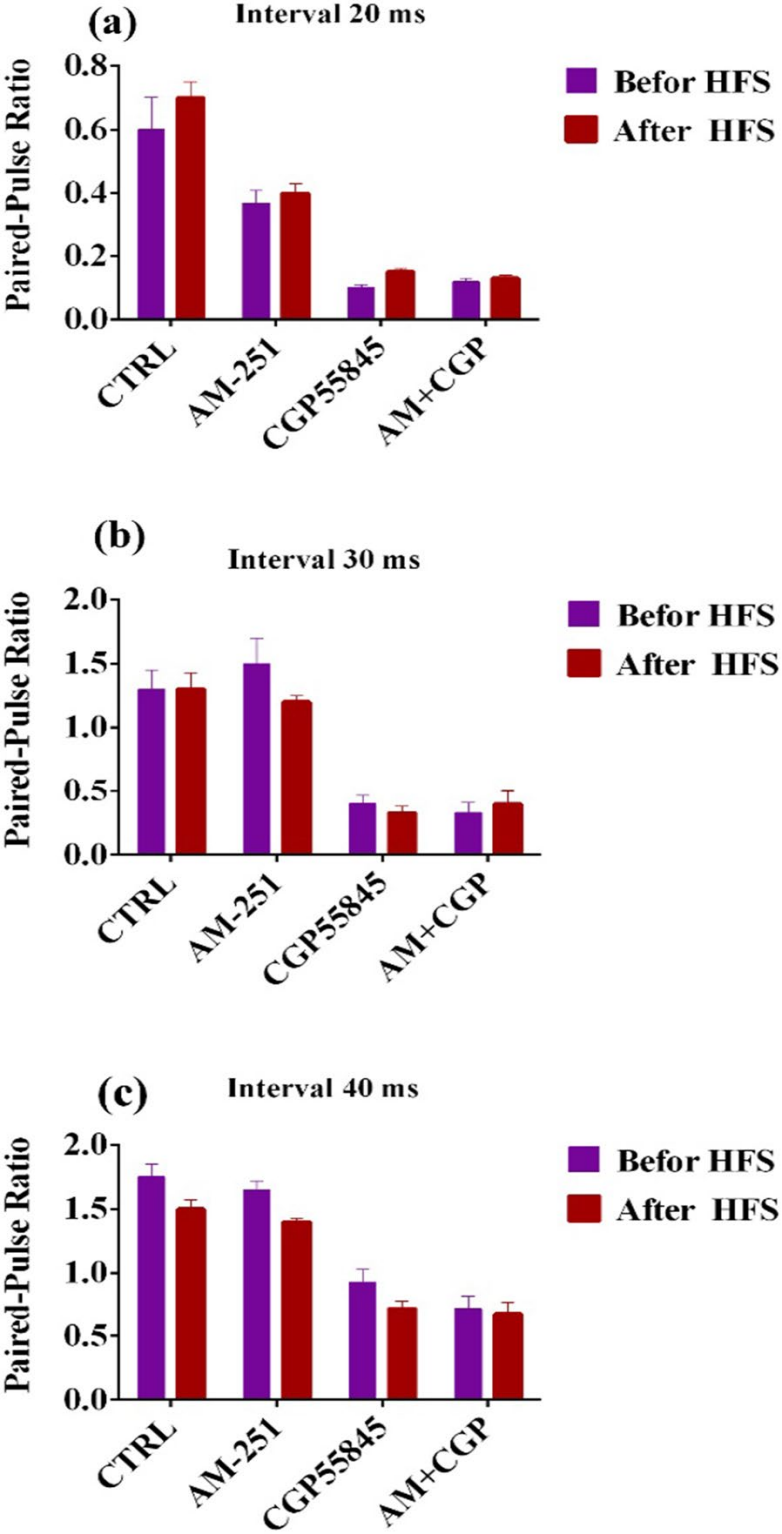
Paired-pulse ratio of evoked field potentials recorded from DG neurons after high-frequency stimulation (HFS) of hippocampal perforant path. The ratios of the first and second field potential amplitudes evoked by paired-pulse stimulation at intervals of 20 (**a**), 30 (**b**), and 40 (**c**) ms have been shown in 4 groups: control, AM-251 (CB1 receptor antagonist), CGP (GABA_B_ receptor antagonist), and AM + CGP. Data were obtained prior to and following the long-term potentiation induction by HFS. Data are reported as mean ± SEM

We compared the amplitude of the second response with the amplitude of the initial response (PPR) after PPS to find the location of LTP in the rats’ DG. Figure 3 indicates the field potentials of the hippocampus evoked by PPS at intervals of 20 (Fig. 4a), 30 (Fig. 4b) and 40 (Fig. 4c) ms prior to and after HFS. Ratios of the second and first field potential amplitudes evoked by PPS at intervals of 20 (Fig. 5a), 30 (Fig. 5b), and 40 (Fig. 5c) ms were plotted for the studied groups. To analyze responses, the first PPR was plotted versus the PPR assessed following LTP induction. PPR was not influenced by LTP induction in the presence of AM251 or CGP55845 either alone or their combination (p > 0.05, Fig. 5). Therefore, the origin of LTP in PP-DG synapses is the postsynaptic neuron and may be mediated through the postsynaptic neurotransmitter receptors.

## Discussion

We evaluated the in vivo impacts of intrahippocampal infusion of CB1 and GABA_B_ receptor antagonists on LTP and STP in the PP-DG pathway of rats. Based on the results of the current study and our previous work [28], AM51 infusion could significantly increase the fEPSP slope and PS amplitude of hippocampal LTP. Conversely, infusion of GABA_B_ receptor antagonist; CGP55845, in the current study, could decrease fEPSP slope and impair LTP induction in the DG. It means that suppression of GABA_B_ receptors using an intra-hippocampal injection of certain antagonists caused LTP impairment in the hippocampal DG. These results are consistent with previous reports. Previous study revealed that the absence of CB1 receptor in cortical glutamatergic neurons increases hippocampal LTP formation, whereas inactivation of CB1 receptor function in forebrain GABAergic neurons leads to reduced hippocampal LTP formation. Furthermore, in CB1-KO, CB1 receptor expression in both glutamatergic pyramidal neurons and GABAergic interneurons is missing, the phenotype is analogous to that detected in Glu-CB1-KO. CB1 receptor antagonism increases hippocampal [17] and prefrontal cortex [40] LTP. The number of pyramidal cells is higher than the GABAergic cells [41, 42]. Furthermore, the computational potency of the pyramidal cells is much higher than the GABAergic neurons, they receive more inputs and target more neurons [23, 43]. Accordingly, CA1 pyramidal neurons receive more excitatory than inhibitory inputs. Therefore, CB1 receptor function in pyramidal cells “overwrites” the function of interneurons in terms of LTP generation.

In a previous report, it has been reported that administration of AM251 (0.2 μM; in DMSO/PBS) inhibits the LTP induction by the stimulus in the hippocampal CA1 area [27]. In addition, it has been demonstrated that endocannabinoids facilitate LTP induction in the hippocampal CA1 pyramidal cells [21]. Conversely, Δ9-tetrahydrocannabinol (Δ^9^-THC) or endogenous cannabinoid agonists administration blocks LTP in the CA1 area in vitro [16, 44], and cannabinoid‐mediated blockade of LTP in the rat hippocampal slice can be reversed by administration of the cannabinoid receptor antagonist [45]. In addition, the cannabinoid‐mediated blockade of LTP was reversed via the facilitation of NMDA receptor function [44]. This may suggest that the AM-251 affected NMDA receptor-dependent form of LTP in the DG.

It has been shown that activation of DG GABA_B_ receptors mainly disinhibited granule cells and reduced the release of GABA from hilar interneurons and enhanced granule cell output [46]. Based on our results it seems that CGP55845 administration disturbs disinhibition on granule cells and consequently GABA release increases. GABA acts on postsynaptic GABA_B_ receptors and leads to prolonged hyperpolarization due to increased K^+^ conductance [47]. Augmentation of the functions of GABA receptor decreases the NMDA excitatory neurotransmission during LTP induction, and consequently impairs LTP [48]. Consistent with our results it has demonstrated that GABA_B_ receptor blockade suppressed hippocampal LTP and impaired spatial learning [49]. In addition, stimulating presynaptic GABA_B_ autoreceptors reduced GABAergic inhibition [46]. Blocking these autoreceptors increased GABAergic inhibition, resulting in impaired LTP. It has been shown that hippocampal GABAergic axon terminals express CB1 receptors [29]. Moreover, according to our results and previous works it seems that cross-talk is possibly associated with balance tuning the GABAergic and endocannabinoid signaling in the hippocampus [29]. In a previous study, it is revealed that GABA_B_ receptor-mediated disinhibition is needed for LTP induction in DG [50]. In the presence of bicuculline as a GABA_A_ receptor antagonist, baclofen (GABA_B_ agonist) failed to enhance the PS [46]. In other words, GABA_A_ receptor antagonist via reduced GABA release could inhibit the disinhibitory effect of GABA_B_ on the PS.

The activation of metabotropic glutamate 5 receptor (mGluR5) and NMDA receptors in dendritic spines [51, 52] at the glutamatergic synapses of the LPP with DG produce the endocannabinoid 2-arachidonoyl-*sn*-glycerol (2-AG) that crosses the synaptic junction and engages CB_1_ receptors on axon terminals to retrogradely reduce release of neurotransmitter during increment of postsynaptic activity [15, 53, 54]. LTP has two major forms: NMDA receptor-independent and NMDA receptor-dependent [55]. Mechanisms underlying the expression of the former form are located presynaptically [55, 56]. In PP-DG LTP, there are two excitatory input pathways, the medial perforant path (MPP) and LPP synapse onto dendrites of DG cells [57, 58]. LTP of the MPP and LPP inputs are blocked by AP5 (an NMDA receptor antagonist), or by the injection of 1,2-Bis (2-aminophenoxy)ethane-N,N,N′,N′-tetraacetic acid (calcium chelating agents) into the postsynaptic cell [58]. This show that LTP in PP-DG pathway is NMDA receptor-dependent [59].

To assess the synaptic location of LTP in the DG, the effect of long-term synaptic plasticity of the hippocampus on the short-term plasticity of the hippocampus after PPS was evaluated. It was also investigated whether the impacts of the CGP55845 and AM51 on LTP can be mediated by the postsynaptic and/or presynaptic partner. PPR was employed for answering this question. Alterations in the paired-pulse responses, measured by the PPR, can be regarded as short-term types of plasticity ranging from milliseconds to seconds. The second response following the PPS of the afferent fibers is depressed or enhanced [60]. PPR was not influenced by LTP induction in the presence of AM251 or CGP55845 either alone or their combination. Therefore, alterations in the PPR are associated with alterations in the transmitter release possibility [60]. With an increase in PPR, there is an increase in the release of the neurotransmitter [60, 61]. GABA_B_ receptors distributed in the CNS are located in pre- and postsynaptic membranes and have disinhibitory and inhibitory impacts and an essential role in neurotransmission [29, 62]. Our observation suggests that CGP55845; specific antagonist for GABA_B_ receptors, significantly impaired LTP in the DG, presumably, at least partly, in the postsynaptic site, because PPR was not influenced by LTP induction in the presence of CGP55845.

One explanation for the lack of change in PPR upon AM251 infusion may be the depression of PPR in response to depolarization. In our study, DG region was stimulated by depolarization to record fEPSPs. Blockade of CB1R activation by AM251 prevented the synaptic depression induced by the postsynaptic depolarization. Therefore, PPR may not be changed by AM251. The depolarization-induced change of PPR prevents the changes in PPR by AM251 [63].

## Conclusion

The site of LTP expression is, at least in part, the postsynaptic site, because PPR was not influenced by LTP induction in the presence of AM251 or CGP55845 either alone or their combination. Therefore, the effect of CB1 and GABA_B_ receptor antagonists on LTP induction at the dentate gyrus of the hippocampus is mediated by postsynaptic changes.

## Data Availability

The data used in our study are available from the authors on reasonable request.
